# INTEGRATING ORAL HYGIENE AND COGNITIVE HEALTH: GLOBAL INSIGHTS AND INTERGENERATIONAL APPROACHES

**DOI:** 10.1093/geroni/igae098.1628

**Published:** 2024-12-31

**Authors:** Xiang Qi, Weiyu Mao, Michele Saunders

**Affiliations:** Chair; Co-Chair; Discussant

## Abstract

The symposium presents four studies investigating the intersection of oral health, cognitive functions, and healthcare accessibility among older adults in China and India. The first study employs a co-design approach to develop a school-based oral hygiene intervention that leverages intergenerational ties between youths and older adults to enhance oral health education. This initiative demonstrates a promising avenue for promoting healthy behaviors across generations, emphasizing family dynamics and resource allocation in the design of oral health interventions. The second review underscores the potential of regular toothbrushing as a simple yet effective strategy to mitigate cognitive decline and dementia risk. This meta-analysis illuminates the direct benefits of oral hygiene on cognitive functions, advocating for the inclusion of oral hygiene practices in daily routines to promote cognitive health. The third study finds significant correlations between cognitive decline and poor oral health using data from the Longitudinal Aging Study in India, highlighting the urgent need for improved dental care accessibility and oral health education as preventive measures against cognitive impairment. Finally, the fourth study investigates into the dental care utilization patterns among older adults in Shanghai reveals a troublingly low engagement with dental services, exacerbated by socioeconomic disparities and insurance coverage limitations. The study calls for comprehensive reforms to extend dental care access and integrate it within broader health insurance schemes. Together, these studies shed light on the critical link between oral health and cognitive health and propose actionable strategies to improve public health outcomes through enhanced education, policy reform, and community-based interventions. Oral Health Interest Group Sponsored Symposium

